# A Study on Consumer-Centric Health Information Provision Strategy Using SWOT-AHP -Focusing on the National Health Information Portal

**DOI:** 10.1007/s10728-024-00505-y

**Published:** 2025-02-04

**Authors:** Jaeeun Baek

**Affiliations:** 1 College of Nursing, Seoul National University, Seoul, Republic of Korea; 2 Department of Data Science Research, Seoul National University Hospital, Seoul, Republic of Korea

**Keywords:** Consumer health information, Health information website assessment, SWOT-AHP, Consumer-centered strategies

## Abstract

Approximately 70% of Koreans access health and medical information online. Health information providers play a crucial role in enhancing public health by ensuring that individuals can effectively consume and utilize this information according to their information-seeking behaviors. However, existing tools for evaluating health information websites have significant limitations. These tools are often one-size-fits-all and lack strategic recommendations for delivering consumer-centered health information. There is a clear need for alternative approaches beyond merely identifying the quality factors that satisfy consumers. A Strengths, Weaknesses, Opportunities, and Threats—Analytic Hierarchy Process (SWOT-AHP) evaluates both internal and external environmental factors of a health information website, which provides strategies based on the prioritization and weighting of quality factors. Specifically, the 'National Health Information Portal,' a platform provided by the Korea Disease Control and Prevention Agency, was assessed through a comprehensive review of prior research and a SWOT analysis, followed by an AHP survey involving 15 experts specializing in health information websites. The findings of the analysis indicate that the most effective development strategy is the SO (Strengths-Opportunities) strategy. This study highlights the need to move beyond uniform evaluation tools and consider the dynamic and complex nature of the Internet, emphasizing the importance of developing prioritized strategies based on evaluations from both consumers and providers.

## Introduction

In contemporary times, there is a noticeable surge in medical consumers turning to the Internet to access health information. This inclination is attributed to the Internet’s convenient, anonymous, and diverse nature, which aligns with the prevailing societal trend of taking charge of one's health and adopting preventive measures against diseases [1]. A survey conducted among South Koreans revealed that the most prominent sources for seeking health information were internet portal sites, blogs, cafes, and national institution websites. These online channels emerged as the most active platforms through which people accessed relevant health-related information [2]. Therefore, Health information providers have a crucial role in promoting public health by assisting individuals in the appropriate consumption and utilization of health-related information based on their search preferences. Specifically, government agencies should play a more dedicated role in this endeavor, as it directly impacts the health and security of the nation. To achieve this objective, the Korean Medical Association and the Ministry of Health and Welfare took a significant step by jointly establishing the National Health Information Portal (http://health.mw.go.kr) in 2011 [3]. It is essential for countries to evaluate the quality of health information to provide reliable data and support citizens in making optimal health decisions [4]. However, current evaluations of health information websites tend to focus solely on identifying the quality of the website and its influencing factors, without providing corresponding strategic recommendations [5–9]. Additionally, while various studies have highlighted the differing perspectives of health information providers and consumers [6, 9–11], existing health information website evaluation methods fail to offer an integrated approach [1, 12]. Therefore, evaluating online health information necessitates an approach that transcends the limitations of traditional evaluation tools by employing a mixed methodology that integrates both qualitative and quantitative assessments. It is crucial to conduct in-depth analyses that uncover strengths and weaknesses, tailored to the specific purpose and platform of the evaluation [13, 14]. Notably, a study demonstrated that combining the Analytic Hierarchy Process (AHP) with interviews to evaluate hospital website quality effectively highlighted and contextualized the discrepancies in metric weighting between consumers and developers [14].

Therefore, this study used the Strengths, Weaknesses, Opportunities, and Threats—Analytic Hierarchy Process (SWOT-AHP) to consider and evaluate the various environments surrounding health information websites from the perspective of consumers who consume health information, and to prioritize strategies accordingly. This study introduces an innovative method for website evaluation that surpasses the limitations of traditional evaluation tools by precisely identifying website issues and formulating strategies to achieve specific goals while improving the website to satisfy consumer needs.

### Literature Review

The HON Code [15], MedCERTAIN [16] and MedCIRCLE [17] which are commonly used for website evaluation, primarily focus on ensuring that a website includes essential elements for credibility from the information provider's standpoint, but they do not evaluate the accuracy of the content itself. The evaluation cannot extend beyond a review of the required elements [18]. DISCERN, a widely used tool for health information research on specific diseases, faces difficulties in remaining recency and may be used by individuals lacking expertise, leading to potential evaluator variability and reduced objective reliability [19]. Meanwhile, Shin and Kim [20] employed the WebQual model to identify 48 consumer-oriented quality factors. Their findings indicate that the provision of health information must account for a lot of factors, such as individuals' needs, behaviors, content quality, health literacy, and information delivery methods. As such, health information should be provided with consideration of the consumer-centered market environment and various influencing factors specific to the digital era, rather than relying solely on the opinions of consumers or experts. Similarly, in a study on CloudIoT-based healthcare (CIoTHC) technology adoption, researchers addressed the limitations of information system-focused studies by taking a holistic approach, analyzing technological, personal, organizational, and environmental factors using the AHP method to provide user-centered strategy [21]. Adepoju research team’s systematic review of website evaluation methods employing Multi-Criteria Decision-Making (MCDM) approaches identified the combination of fuzzy logic and AHP as the most frequently used method [12]. However, most studies merely identify factors that can serve as evaluation indicators by measuring their weights, without advancing to the development of strategic models that extend beyond the evaluation process [9]. As a weight measure, there is a tendency to prioritize theoretical models over practical realities. Moreover, evaluations have typically concentrated on specific quality criteria, neglecting a comprehensive consideration of various factors [12]. A critical study of the AHP evaluation method suggested that decision makers’ relative weightings might be a starting point for advancing MCDM models, as the AHP method may sometimes diverge from the intuitive decisions made by decision-makers [22].

To address these limitations, this study employed the SWOT-AHP method. The SWOT-AHP method is predominantly applied in business management strategies, with its effectiveness demonstrated in applications such as the SWOT-AHP model used by Starbucks, a leading global coffee retailer [23]. In the medical field, recent studies have employed SWOT-AHP to offer a comprehensive view of the current status of implants [24] and to determine the development direction and strategy for home-based exercise programs [25].

The strengths of this study, which utilizes the SWOT-AHP approach to overcome the limitations of traditional website evaluation methods, include the following. First, SWOT-AHP enables the measurement of the overall quality of health information websites by considering both internal and external environmental factors of the project [26]. Unlike traditional tools that may focus on a limited range of quality attributes, SWOT-AHP offers a more comprehensive evaluation. Second, many studies employing the AHP have predominantly assessed services from the perspective of health information providers. However in this study, the author has asked experts who understand what consumers want to evaluate websites using AHP. Furthermore, the author integrated the SWOT-AHP results with expert opinions on strategies for developing consumer-centered health information websites and proposed targeted, actionable strategies. This approach allows for the suggestion of strategies to develop websites that cater to consumer needs.

Consequently, the study offers a novel strategic approach for analyzing and evaluating health information websites, emphasizing the prioritization of strategies derived from thorough evaluations. In particular, it proposes a new paradigm for health information website assessment by integrating comprehensive perspectives from both consumers and experts, thus advancing the current evaluation practices.

## Methods

### SWOT Analysis

SWOT analysis is a longstanding and extensively employed tool for developing strategies [27].

The fundamental principle of SWOT analysis involves identifying strengths and weaknesses from internal capabilities, and opportunities and threats from external environmental factors. The purpose is to formulate specific strategies that leverage internal capabilities to address environmental challenges. Instead of focusing solely on resources and capabilities, it is crucial to pinpoint complementary capabilities to effectively address environmental factors [26]. The SWOT analysis process encompasses several stages, including agenda and topic selection, internal environment analysis, external environment analysis, opinion collection and strategy formulation, strategy implementation, and feedback [27]. Based on the SWOT analysis, four strategies—SO (Strengths-Opportunities), ST (Strengths-Threats), WO (Weaknesses-Opportunities), and WT (Weaknesses-Threats)—can be formulated by pairing internal factors (Strengths and Weaknesses) with external factors (Opportunities and Threats) [26].

#### AHP

The AHP is a systematic approach that involves breaking down a complex problem into smaller subproblems and then aggregating the solutions of these subproblems to form a cohesive conclusion [28]. Widely used across various disciplines, AHP has been employed as a pre-feasibility study method in more than 500 projects since 2000. Moreover, decision-making involves both quantifiable attributes (those that can be measured) and psychological attributes (ideas, feelings, and societal beliefs), and AHP is recognized as a robust measurement tool that addresses both areas effectively [29].

#### SWOT-AHP

**Fig. 1:**
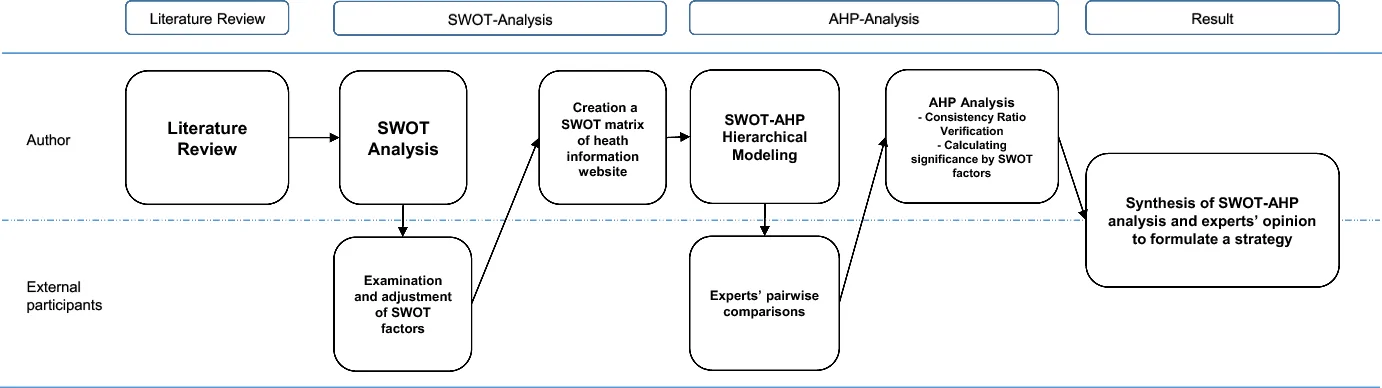
The Study Framework. The application process of SWOT-AHP involves four main steps

Figure 1 outlines the framework of this study, detailing the processes of SWOT analysis and AHP analysis. This framework specifies the role of research external participants in ensuring objectivity by providing a design aimed at reducing the bias of the author.

**Fig. 2:**
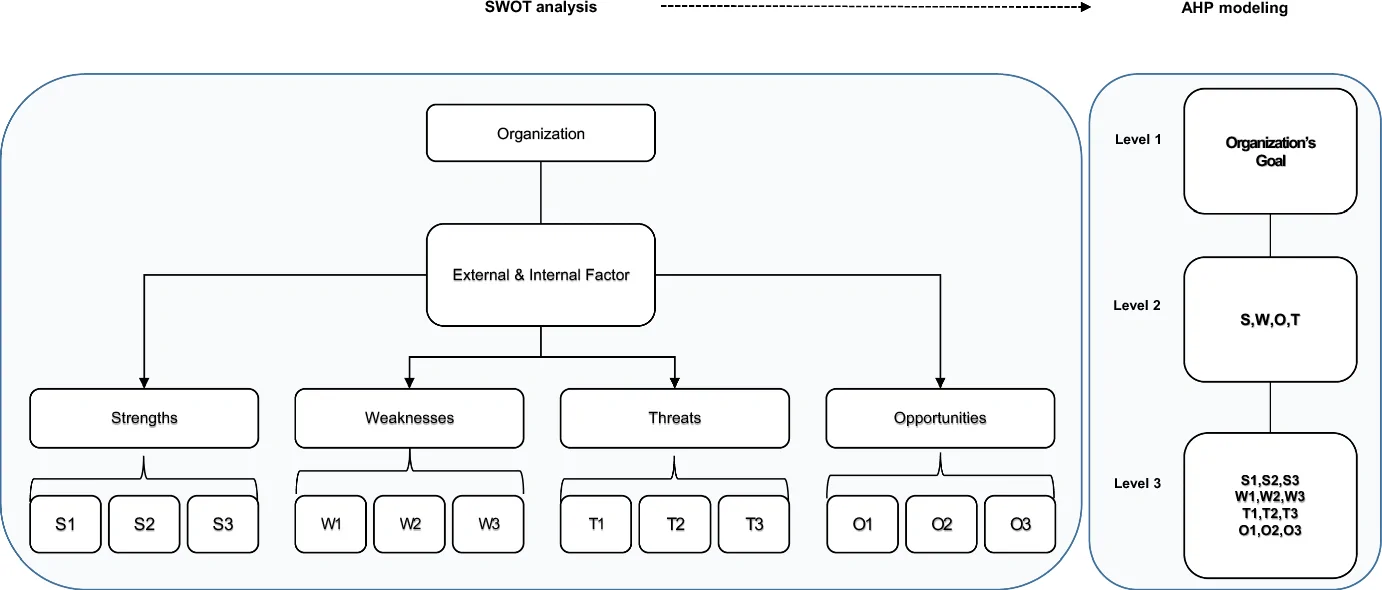
The Step 1 of SWOT-AHP

*Step 1*: Fig. 2 illustrates the flow of Step 1. Define the decision problem's goal and establish a decision hierarchy by organizing subordinate elements into a structured hierarchy to achieve this goal. In the SWOT-AHP methodology, SWOT analysis is employed to verify the details of goal setting and hierarchical structure. The AHP model is subsequently developed based on the results of the SWOT analysis. Typically, the objective of the SWOT analysis is placed at the first hierarchical level, each SWOT element necessary for achieving this objective comprises the second level, and the detailed components of each SWOT element form the third level [23].

*Step 2*: Collect judgment data by conducting pairwise comparisons of items within each hierarchical tier. To facilitate quantitative judgment through these comparisons, a reliable and consistent scale is required. The 9-point scale, illustrated in Table 1, is the most used method for this purpose. This scale was proposed by Saaty and is prevalent in most AHP applications. When there are *n* elements, a total of $\frac{\mathrm{n}(\mathrm{n}-1)}{2}$ pairwise comparisons are required [29].

**Table 1:** The 9-point scale

1	3	5	7	9	2, 4, 6, 8
Equal importance	Moderate importance	Strong importance	Very Strong or demonstrated importance	Extreme importance	The median of the values presented

Saaty, T. L. (2005). (Network Analytical) Decision Making. Seoul: *Donghyeon*. http://snu-primo.hosted.exlibrisgroup.com/82SNU:82SNU_INST21442099830002591

To estimate the relative weights of the decision factors, a pairwise comparison matrix is constructed. For a single level, assuming that the number of comparison elements is *n* and the relative importance is X_ij_(i, j = 1,2,3 ~ n), Matrix X is a positive reciprocal square matrix, where X_ij_ is the comparison between element i and j. Naturally, X_ji_ = $\frac{1}{\mathrm{xij}}$.

As an example, a pairwise comparison matrix X with 4 elements is presented below:

**Table:** 

X =	X1	X2	X3	X4
X1	1	X_12_	X_13_	X_14_
X2	$\frac{1}{\mathrm{x}12}$	1	X_23_	X_24_
X3	$\frac{1}{\mathrm{x}13}$	$\frac{1}{\mathrm{x}23}$	1	X_34_
X4	$\frac{1}{\mathrm{x}14}$	$\frac{1}{\mathrm{x}24}$	$\frac{1}{\mathrm{x}34}$	1

*Step 3*: Validate the survey results through AHP by calculating the Consistency Index (CI) and Consistency Ratio (CR) after performing pairwise comparisons. The key objective is to assess the extent to which raters maintain transitive consistency—a coherent evaluation of the priority relationships among items within the stratification analysis process. Transitive consistency is evaluated using the CI and the CR, where the latter incorporates a random index (RI) derived from the data. The CI is calculated using λ max, the largest eigenvalue of the pairwise comparison matrix, and N, the number of elements within the matrix. For a perfectly consistent comparison matrix, λ max = *N*, and the inequality λmax ≥ *N* is always satisfied [30]. The RI varies with different values of *N* and is detailed in Table 2 [31]. According to Saaty, a CR value of 0.1 or lower is generally acceptable, indicating a high level of consistency. However, CR values up to 0.15 are considered reasonable, with the maximum threshold often set at 0.2 or less for acceptable consistency [32]. A common maximum acceptable CR value is 0.15.

**Table 2:** Random Index^a^

N	1	2	3	4	5	6	7	8	9	10
RI	0.00	0.00	0.58	0.90	1.12	1.24	1.32	1.41	1.45	1.49

^a^Saaty, T. L. (1990). How to make a decision: the analytic hierarchy process. *European journal of operational research*, 48(1), 9–26.https://doi.org/10.1016/0377-2217(90)90057

The Consistency Ratio (CR) is calculated as follows: CR = $\frac{\mathrm{CI}}{RI}$

The formula for CI is: CI(X) = $\frac{{\lambda {\mathrm{max}} - {\mathrm{N}}}}{N - 1}$

*Step 4*: Synthesize the relative weights of the factors within the hierarchy to obtain an overall ranking of the alternatives for decision-making. This process is accomplished by aggregating the weights across each tier of the hierarchy. Specifically, it involves multiplying the relative weights of elements in the lower tier by the relative weights of the corresponding elements in the upper tier [29, 33]. For instance, if the weights of SWOT elements are denoted as α, β, γ, δ and the weights of all factors within each SWOT category are labeled as a, b, c…, l, then the composite weight for each factor is calculated as (α × a), (β × b), …, (δ × l). The final overall ranking is derived by summing these composite weights (Table 3).

**Table 3:** Comprehensive Importance Degree

Second level’s factor weights		Third level’s factor weights		Comprehensive factor weights
S	α	S1	a	α x a
		S2	b	α x b
		S3	c	α x c
W	β	W1	d	β x d
		W2	e	β x e
		W3	f	β x f
O	γ	O1	g	γ x g
		O2	h	γ x h
		O3	i	γ x i
T	δ	T1	j	δ x j
		T2	k	δ x k
		T3	l	δ x l

### Participants

Park reported that in preliminary feasibility studies conducted by government organizations, AHP analysis typically involves eight evaluators [32]. A composite rating is derived from the responses of six evaluators, excluding the evaluator who provided the maximum and minimum values. This study incorporates both service provider and consumer perspectives and derives results. For this purpose, 15 participants were selected as research subjects, and results were derived by including all evaluators' responses, including those with the maximum and minimum values. The participants of the study were selected based on their expertise in fields related to health information websites, with each subject possessing more than five years of experience in research, education, and business within the relevant domain.

### Data Collection

The SWOT analysis in this study was derived from analyzing various sources of prior research on consumer health information provision, research conducted by government organizations focusing on health information dissemination, and research reports pertaining to the National Health Information Portal, which is currently operated by the Korea Centers for Disease Control and Prevention [34–40].

To gather data about the experts, the researcher utilized an online questionnaire. A survey request email was sent to a total of 24 individuals over 20 days, from March 30, 2023, to April 10, 2023. Out of these recipients, 15 subjects responded to the survey. The AHP survey responses were collected using the 'I make it' program [41], a specialized software designed for conducting AHP assessments.

### Ethics Statement

Electronic informed consent was obtained, and the study was approved by the Institutional Review Board (IRB) of Seoul National University Hospital (IRB No. C2303-117–1413). This article is modified from the author's master's thesis.

## Results

### Participant’s Characteristics

Of the total 15 study subjects, 27% were male and 73% were female. The age distribution of the participants was as follows: 7% were in their 20 s, 33% in their 30 s, 47% in their 40 s, and 13% in their 50 s. By specialty, the top three areas of expertise are health information generation or evaluation (19%), health education (14%), health promotion and disease prevention (13%). About 60% of our experts are from the consumer field, professionals in UX/UI design, media, health education, consumer studies, website development and management, and communications, providing insights that reflect the voice of the consumer (Table 4).

**Table 4:** Participant’s characteristics

Participants		Frequency	Percentage(%)
Sex	Male	4	27
	Female	11	73
Age	20 s	1	7
	30 s	5	33
	40 s	7	47
	50 s	2	13
Areas of expertise	Create or Evaluate Health Information	7	19
	Health Promotion and Disease Prevention	5	13
	UX/UI design	3	8
	Media	2	5
	Health Education	5	14
	Health Policy	3	8
	Consumer Studies	3	8
	Journalism	0	0
	Website Development and Management	3	8
	Communications	5	14
	HR Consulting/System Implementation	1	3
Career	5 years or less	0	0
	5 to 10 years	8	53
	10 to 15 years	4	27
	15 years or more	3	20
Occupation	Professors	5	33
	Consumer-advocate	3	20
	UX/UI Designer	1	7
	Programmer	2	13
	Student researcher (PhD)	2	13
	Doctor	1	7
	Health information image designer	1	7

### SWOT Analysis

**Fig. 3:**
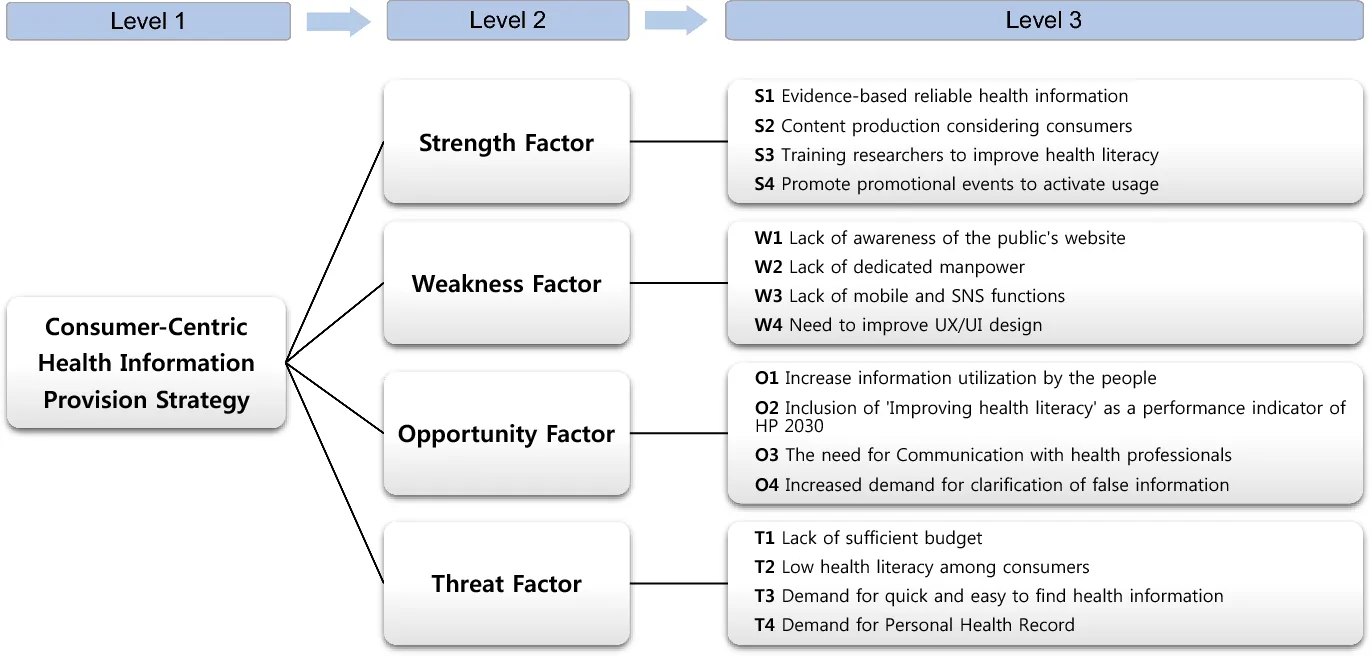
SWOT-AHP Model

Systematic thinking was applied to ensure objectivity and prevent any potential bias from the authors when formulating the SWOT-Matrix. The final SWOT matrix was then developed by three members of the National Health Information Portal research team, reflecting feedback on the SWOT matrix [26]. The result of the SWOT analysis, SWOT-matrix was derived. From the SWOT analysis, a total of 11 strengths, 10 weaknesses, 7 opportunities, and 10 threats were identified. To simplify the AHP modeling process, these factors were further summarized into four main factors to guide the development strategy for the National Health Information Portal (Table 5). The AHP model is composed of three layers, the first level is the purpose of the study, the second level is the four factors of SWOT, and the third level is each of the four factors in S, W, O, and T, totaling 16 factors. The SWOT-AHP model is the same as Fig. 3.

**Table 5:** SWOT Matrix of the National Health Information Portal

S	Evidence-based reliable health information	Evidence-based reliable health information
	Cooperation with government, consumers, academic organizations, and search engine companies	Content production considering consumers
	Providing core health practice messages	Training researchers to improve health literacy
	Content production considering consumers	Promote promotional events to activate usage
	Adoption of programs for user analysis	
	Promote promotional events to activate usage	
	Periodic evaluation and reproduction of health information	
	Establish development strategies to overcome problems	
	Offer content linkage with relevant domestic agencies	
	Training researchers to improve health literacy	
	Designing knowledge-based health information	
W	Lack of awareness of the public's website	Lack of awareness of the public’s website
	Lack of dedicated manpower	Lack of dedicated manpower
	Lack of consumer-centered health information experts	Lack of mobile and SNS functions
	Lack of sufficient writing time	Need to improve UX/UI design
	Lack of mobile and SNS functions	
	Need to improve UX/UI design	
	Inconvenient search function	
	Poor speed of website	
	Lack of health information for low-health literacy level groups	
	Frequent errors in the content management system	
O	Demand for information based on user analysis	Increase information utilization by the people
	Increased demand for clarification of false information	Inclusion of 'Improving health literacy' as a performance indicator of HP 2030
	Increase information utilization by the people	The need for Communication with health professionals
	Increased Internet usage rate among citizens	Increased demand for clarification of false information
	Inclusion of 'Improving health literacy' as a performance indicator of HP 2030	
	The need for Communication with health professionals	
	Supporting cooperation in search engine advancement	
T	Lack of sufficient budget	Lack of sufficient budget
	Low health literacy among consumers	Low health literacy among consumers
	Demand for quick and easy to find health information	Demand for quick and easy to find health information
	Demand for healthcare utilizing smart technology	Demand for Personal Health Record
	Demand for Personal Health Record	
	Lack of Korean health literacy measurement tools	
	Diverse socio-cultural backgrounds of users	
	Need for rapid information on new diseases	
	Demand for integrated information beyond just diseases	
	Increased utilization of mobile and SNS

### AHP Analysis

The experts conducted a pairwise comparison analysis of the four factors in the second layer and each of the four factors in S, W, O, and T as the third level [32, 41]. According to standard principles, AHP pairwise comparisons require conducting six comparisons for each factor. However, to reduce the burden on experts, the author employed the ‘I make it’ program, an AHP application developed to decrease the number of pairwise comparisons while employing parallel logic to ensure equivalent effectiveness. As a result, I was able to conduct a total of five pairwise comparisons following the formula '2n-3' (where n represents the number of pairwise comparison factors), as depicted in Table 6 [42]. The comprehensive results of these five sets of pairwise comparisons involving 15 participants are presented in Tables 7, 8, 9, 10 and 11.

**Table 6:** Pairwise comparison set

Level		set	Pairwise comparison set					
Second		1	S:W	S:O	*S:T	W:O	W:T	O:T
Third	S factor	2	S1:S2	S1:S3	⁎S1:S4	S2:S3	S2:S4	S3:S4
	W factor	3	W1:W2	W1:W3	⁎W1:S4	W2:W3	W2:W4	W3:W4
	O factor	4	O1:O2	O1:O3	⁎O1:O4	O2:O3	O2:O4	O3:O4
	T factor	5	T1:T2	T1:T3	⁎T1:T4	T2:T3	T2:T4	T3:T4

^*^Omitted pairwise comparisons

**Table 7:** Pairwise comparison of SWOT factors

SWOT	S	W	O	T	Importance Degree	CR
S	1	5/2	10/7	-	0.375	0.00208
W	2/7	1	5/9	7/9	0.153	
O	7/10	9/5	1	9/8	0.258	
T	-	9/7	8/9	1	0.213

**Table 8:** Comparison Matrix of Strengths Group

Strengths	S1	S2	S3	S4	Importance degree	CR
S1	1	2	4	-	0.5264	0.00294
S2	1/2	1	17/7	17/5	0.272	
S3	1/4	7/17	1	3/2	0.1222	
S4	-	5/17	2/3	1	0.0794

**Table 9:** Comparison Matrix of Weaknesses Group

Weakness	W1	W2	W3	W4	Importance degree	CR
W1	1	10/3	3	-	0.5041	0.00013
W2	3/10	1	1	8/9	0.1536	
W3	1/3	1	1	1	0.1701	
W4	-	9/8	1	1	0.1723

**Table 10:** Comparison Matrix of Opportunities Group

Opportunity	O1	O2	O3	O4	Importance degree	CR
O1	1	26/9	11/5	-	0.4648	0.00016
O2	9/26	1	5/7	1	0.1572	
O3	5/11	7/5	1	4/3	0.2144	
O4	-	1	3/4	1	0.1636

**Table 11:** Comparison Matrix of Threats Group

Threat	T1	T2	T3	T4	Importance degree	CR
T1	1	1	1	-	0.223	0.00237
T2	1	1	8/9	2/3	0.2148	
T3	1	9/8	1	3/5	0.2202	
T4	-	3/2	5/3	1	0.3421	

**Fig. 4:**
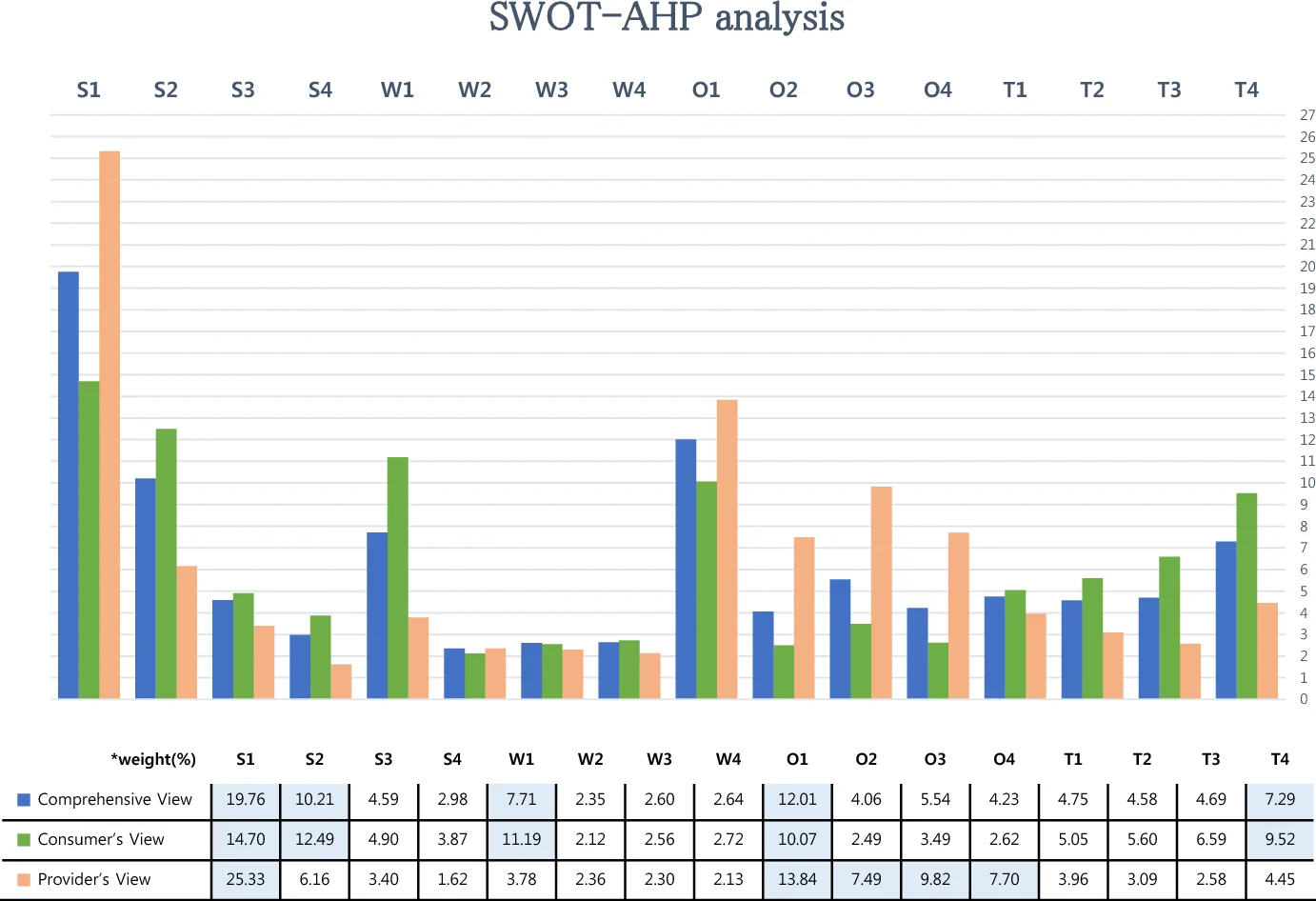
SWOT-AHP analysis results

The comprehensive SWOT-AHP results are presented in Table 12 and Fig. 4. In the SWOT-AHP, each factor within the S, W, O and T categories has been given an L-weight, which indicates its relative importance within its respective group. This weight allows us to understand the significance of each factor compared to others within the same category. G-weight represents the relative importance of all factors reflecting the weight of the second layer. According to Table 3, the G-weight is calculated by multiplying the L-weight by the weight of the Group. By considering both L-weight and G-weight, the author planned effectively the development strategy and prioritized to creation consumer-centered health information platform. Within AHP, the CR serves as a crucial metric for assessing response reliability. In this study, all 15 experts exhibited CR meeting the prescribed criterion of 0.1 or lower. This outcome confirms the meticulous maintenance of logical consistency throughout the analysis process.

**Table 12:** Comprehensive SWOT-AHP results

Internal factors				
Strength, 0.375	L-weight	G-weight	Rank	CR
S1 Evidence-based reliable health information	0.5264	0.1976	1	0.00294
S2 Content production considering consumers	0.272	0.1021	3	
S3 Training researchers to improve health literacy	0.1222	0.0459	9	
S4 Promote promotional events to activate usage	0.0794	0.0298	13	

Weakness, 0.153	L-weight	G-weight	Rank	CR
W1 Lack of awareness of the public's website	0.5041	0.0771	4	0.00013
W2 Lack of dedicated manpower	0.1536	0.0235	15	
W3 Lack of mobile and SNS functions	0.1701	0.026	14	
W4 Need to improve UX/UI design	0.1723	0.0264	13	

External factors				
Opportunity, 0.258	L-weight	G-weight	Rank	CR
O1 Increase information utilization by the people	0.4648	0.1201	2	0.00016
O2 Inclusion of 'Improving health literacy' as a performance indicator of HP 2030	0.1572	0.0406	12	
O3 The need for Communication with health professionals	0.2144	0.0554	6	
O4 Increased demand for clarification of false information	0.1636	0.0423	11	

Threat, 0.213	L-weight	G-weight	Rank	CR
T1 Lack of sufficient budget	0.223	0.0475	7	0.00237
T2 Low health literacy among consumers	0.2148	0.0458	10	
T3 Demand for quick and easy to find health information	0.2202	0.0469	8	
T4 Demand for Personal Health Record	0.3421	0.0729	5

As a result of the relative weight analysis of each SWOT factor, the priorities were determined in the following order: S, O, T, and W. The respective weights assigned to these priorities were 0.375, 0.258, 0.213, and 0.153. Based on the findings from the SWOT-AHP comprehensive analysis, the optimal development strategy identified for consumer-centered health information websites is the SO strategy. This strategy capitalizes on the strengths of Evidence-based reliable health information (S1, 0.1976) and Content production considering consumers (S2, 0.1021). To seize the opportunities that increased information utilization by the people (O1, 0.1201) and the need for Communication with health professionals (O3, 0.0554). In addition, it is necessary to supplement the Weaknesses of the public's website’s lack of awareness (W1, 0.0771) and to come up with strategies for the demand for Personal Health Record (T4, 0.0729). Meanwhile, in terms of weights, L-weight and G-weight were ranked differently. Within each S, W, O, T of attributes in the second level, S1 (0.5246), W1 (0.5041), O1 (0.4648), and T4 (0.3421) are the first order of weight. The absolute value of the weights shows that O1 was lower than that of W1, but in the G-weight, which reflects the weight of the group, O1 has the second-highest weight. These findings indicate that solely prioritizing a project based on the importance of specific quality factors on a health information website may not lead to an optimal business operation. Therefore, the research confirms the necessity of evaluating the health information website, considering both internal and external project environments. The strategy should be established by prioritizing according to the evaluation results.

The experts were divided into two groups: 60% of the participants were experts from the consumer perspective, while the remaining 40% were experts from the provider perspective. Subsequently, the analysis results from both perspectives were compared to gain insights into the differing viewpoints and considerations of each group.

From the consumer perspective, the analysis results indicated higher weights in the following order: S, T, O, and W, with corresponding weights of 0.359, 0.267, 0.186, and 0.185, respectively. In comparison to the overall results, the highest importance of the strength factor remained consistent, reaffirming its significant impact from the consumer viewpoint. The ranking of the top five factors (S1, S2, W1, O1, and T4) changed, but the components remained the same.

From the provider's perspective, the analysis results showed higher weights in the following order: O, S, T, and W, with corresponding weights of 0.3884, 0.365, 0.14, and 0.105, respectively. According to the experts' viewpoint, the opportunity factors were given the highest priority, closely followed by the strength factors. In addition, considerable weight was given to the S1 factor, and all the second to fourth-ranked factors were opportunity factors (O1, O2, O3, and O4).

The analysis reveals that the utmost priority for health information lies in its reliability, as evidenced by "Evidence-based reliable health information" consistently ranking as the top first factor from both the consumer and provider expert's perspectives, as well as in the comprehensive viewpoint encompassing both consumers and providers. Additionally, the opportunity to "Increase information utilization by the people" consistently appeared among the top five factors.

### Expert’s Opinion

Finally, to address specific aspects not encompassed by the AHP elements and to enhance the findings from the SWOT-AHP analysis, the author gathered expert opinions online regarding strategies for the operation and management of consumer-centered health information websites (Table 13). The author classified the keywords extracted from the free opinions provided by the experts according to the "Online Consumer Information Evaluation Framework" presented in the "Research on Measures for Improving Online Provision of Consumer Information" [43]. The analysis revealed that experts expressed the most opinions concerning the ‘Understandability’ in the area of information quality and the ‘Easy of accessibility' associated with system quality.

**Table 13:** Expert’s opinion in the area of evaluating online consumer information

Evaluation factors	Assessment areas	Sub-areas		Expert’s opinion
Information Quality	Information Content	Accuracy		Fundamental quality health information
		Diversity		-
		Reliability		Trustworthy information
		Understandability		Understandable / From the perspective of the people/Easy term and image for consumer/ Health information at the consumer's level/ Processing information for consumers /Health literacy
		Up-to-date		Seasonal health information and preventive vaccinations. / Timeliness and Promptness / Timely and Appropriate Information
		Connectivity		-
System Quality	Design and interface	Conveniences	Ease of accessibility	Easy Accessibility / Promotion and Marketing Strategy / Ease of Accessing Information/ Seems to be Less Known/ Usability/ Portal Promotion/ Access to Consumers/ Awareness/ Website Ranking/ Portal Awareness/ Lack of Promotion
		Communicability	Search usability	-
			Readability	Text Readability is Poor
			Changes based on user behaviors	-
			Consistency of design	Menu Structure and UI Should Be Convenient
			Intuitiveness of design	UI and UX Design/ I'm not sure if there's more meaning beyond gathering a lot of information in one place
	System Operations and Management	Stability	Automation	Channels like KakaoTalk/ Automation
			Security	-
			Optimization	-
Service Quality	Service	Reactivity	The speed of questions and answers	-
			Diversity of feedback channels	Consumer Channels/ Diverse Channels/ Incorporating Consumer Opinions
			Personalization by audience	For elderly population
		Flexibility	Easy navigation	No description on the website/ Difficult to understand what the website is about
			Degree of openness	-
			accessibility	-
		Integration	Interoperability	-
			Unify information provision	Mobile App Development/ Mobile App

Lee, Bohan, & Lee, KumNoh (2022). Research on Measures for Improving Online Provision of Consumer Information. Korean Consumer Agency, from: https://www.kca.go.kr/home//sub.do?menukey=6101&mode=view&no=1003469456

### Integrated Results

Rather than relying solely on the frequency of expert opinions and the weight of AHP results to determine the prioritization of projects, the author synthesized the SWOT-AHP outcomes with expert opinions to formulate systematic and practical strategies. Firstly, to strengthen people's motivation to use health information, the website should emphasize its strengths by prominently displaying explanations on "the importance of health information" and its role as a platform providing "reliable health information based on evidence" and "health information considering consumers." Second, it is crucial to implement a counseling window within the website to address the growing demand for health information utilization and facilitate communication with health experts. Due to the low weight of mobile and Social Network Service (SNS) functions, communication channels are needed within the website rather than setting up a separate communication field, which will not only satisfy consumers' needs but also increase the website's awareness. Considering the demand for 24-h access to information, relying on round-the-clock waiting and counseling by health professionals may lead to resource inefficiencies. To address this, a more effective alternative would be to develop and provide reliable interactive AI to consumers. Third, a long-term public relations strategy should be developed to address the lack of public awareness regarding the website. This strategy involves Search Engine Optimization (SEO) to improve the national health information portal's visibility and search rankings. Additionally, the previously mentioned counseling window and consumer-oriented content production strategies can be instrumental in enhancing consumers' perception of the website and encouraging active communication. Fourth, in implementing the strategy for providing Personal Health Records (PHR), it is essential to consider the limitations of the current PHR system [44]. To overcome these limitations, the utilization of the Open Application Programming Interface (API) effectively is recommended. Health centers and examination institutions should proactively expose health information from the national health information portal to the public. Public health centers and examination institutions play a pivotal role in disease prevention and health promotion within the community. Particularly, public health centers can cater to the needs of all citizens, including vulnerable populations. Collaborating with local public health centers and guiding examination institutions to use the portal's health information when notifying examination results can foster health promotion and disease prevention behavior among the public.

## Discussion

This study employed the SWOT-AHP method as a novel website evaluation approach, aiming to address the limitations of existing website evaluation tools and provide an optimal development strategy based on the evaluation results. The research holds significant importance in offering a comprehensive and well-informed analysis of the internal and external environment of the health information website. The research findings strongly advocate against superficial evaluations using a standardized tool for all websites.

Based on the results of this study, we discuss the following. First, the study's findings highlighted the inadequacy of using evaluation tools with identical criteria to assess health information websites. In the SWOT-AHP analysis, it was observed that factors related to health literacy, a prominent research trend in online health information, were prioritized but assigned relatively low weightings. Specifically, the factors "Educating researchers to improve health literacy (S3)", "Low health literacy of consumers (T3)" and "Including health literacy as a performance indicator of HP 2030 (O2)" were ranked 9th, 10th, and 12th, respectively, among the overall factors. On the other hand, in a study examining the usage of a national health information portal, the research team demonstrated that the usability and visualization of information significantly influenced users' willingness to revisit the portal and recommend it to others [45]. Nevertheless, it is notable that factors like UX/UI design, mobile apps, and social media—key components that significantly impact usability for consumers with low health literacy [46] —were weighted lower. These results imply that the evaluation results of websites considering complex environmental factors differ markedly from those achieved through conventional tools. Therefore, the National Health Information Portal should concentrate on enhancing other facets of the website rather than focusing solely on improving health literacy and usability for consumers. Despite being a health information website intended for public use, the National Health Information Portal must prioritize strategies that address the public's lack of awareness of the website as a source of evidence-based and reliable health information, as well as overcome its current inability to highlight its strengths effectively. To achieve this, it is essential to enhance the National Health Information Portal by conducting an in-depth analysis and comparison of websites that excel in presenting reliable and trustworthy information. Upon reflection, conducting a consumer assessment is crucial to reimagine their needs and identify any obstacles they may face.

Second, since there was a clear difference in the perspective of consumers and providers, we should pay attention to the evaluation of health information websites that confirm opinions only from consumers or providers. Despite the clear differences between the perspectives of information consumers and providers, 'trustworthiness' and 'usability' emerged as common priorities, highlighting the need for targeted strategy development and additional research. Health information for the public, which uses "Reliability" as the standard for information selection, has confirmed that government-led information with public confidence is needed, but it is necessary to think about the limitations that are still difficult to access the information. According to the Korea Consumer Agency's announcement in 2016 [47], health-related consumer information in the public sector of Korea is prioritized for the establishment of reliable data and the production of information. That study revealed while the data is presented resembling an encyclopedia of simple information, its practical value as "Information" that can be effectively utilized is not assured. Also, the results from the 2021 "Health Literacy and Policy Implications from the Korean Medical Panel" further support the call for a new paradigm in health information, as they reveal a notable deficiency in health information access and utilization by the public [48]. Rocha suggests developing a methodology for improving website quality that integrates insights from both experts and users [49]. Thus, the author hope that this study will serve as a catalyst for future research that evaluates health information websites from a comprehensive perspective and contributes to the development of effective improvement strategies by conducting in-depth and novel evaluations.

## Conclusion

The comprehensive evaluation of the national health information portal using the SWOT-AHP method revealed that the SO strategy is the most suitable approach. This strategy considers both the health information provider's perspective and the consumer's perspective, emphasizing the significance of 'Reliability' and 'Availability' as essential quality factors in health information provision.

The development strategies proposed for the National Health Information Portal are as follows: (1) providing clear statements regarding its provision of evidence-based and reliable health information to boost user motivation,

(2) creating a communication channel and raising awareness through the addition of a consultation window on the portal, (3) utilizing the Open API to enable various organizations to access and use health information [50, 51] and (4) recommended to enhance the visibility of the National Health Information Portal through search engine optimization (SEO) and to improve consumer awareness [52].

This study identifies previously unrecognized issues and proposes strategies for improving the effectiveness of health information dissemination. However, there are limitations to this study. First, it does not account for cultural diversity in the context of a website accessed by individuals from various nationalities in Korea. Future research should consider providing information in multiple languages and evaluate these aspects to address this limitation.

Second, we could not identify the highly specific factors of quality because we controlled four sub-factors of SWOT in the model for consistent responses of AHPs. Since the usability of information was prioritized by both consumers and experts in the study, it is necessary to check the prioritization of usability-oriented details.

However, if Korea's National Health Information Portal focuses on implementing priority strategies identified in this study and supports specific initiatives such as UX/UI design improvements, and the utilization of mobile and social media—areas of primary concern for consumers—it can significantly contribute to achieving the objectives of Korea's Health Plan 2030. Additionally, measuring the health literacy of consumers who consume the built health information and developing customized consumer health information based on it will promote health promotion and disease prevention.

First of all, the study suggests a comprehensive approach to evaluating various websites using the SWOT-AHP method to derive effective strategies. By synthesizing and analyzing multiple results, the study provides valuable insights that can serve as crucial research data for establishing a consumer-centered health information website, tailored to the unique needs and preferences of users.

As a follow-up study, we propose applying this methodology to evaluate websites in other fields and plan to develop an explainable AI algorithm capable of conducting SWOT-AHP evaluations. This algorithm will be based on accumulated evaluation results, internal reports, user satisfaction metrics, and website data such as SEO and Google Analytics. While the automation of website evaluation represents a forward-looking approach, it must integrate expert opinions that consider consumer needs and complex environments in addition to AI-driven assessments [53].
