# Correction: A Study on Consumer-Centric Health Information Provision Strategy Using SWOT-AHP-Focusing on the National Health Information Portal

**DOI:** 10.1007/s10728-025-00515-4

**Published:** 2025-04-11

**Authors:** Jaeeun Baek

**Affiliations:** 1 College of Nursing, Seoul National University, Seoul, Republic of Korea; 2 Department of Data Science Research, Seoul National University Hospital, Seoul, Republic of Korea

**Correction to: Health Care Analysis** **https://doi.org/10.1007/s10728-024-00505-y**

In the original publication of the article Tables 6, 12 and 13 contain errors.

In Table 6, the header in column 2 was incorrectly labelled “set”. But should have been blank. Consequently, the original “set” and “Pairwise comparison set” should be shifted one column to the right.

The correct and incorrect versions of Table 6 are shown below:

The incorrect version of Table 6 is

**Table 6:** Pairwise comparison set

Level	set	Pairwise comparison set						
Second		1	S:W	S:O	*S:T	W:O	W:T	O:T
Third	S factor	2	S1:S2	S1:S3	*S1:S4	S2:S3	S2:S4	S3:S4
	W factor	3	W1:W2	W1:W3	*W1:S4	W2:W3	W2:W4	W3:W4
	O factor	4	O1:O2	O1:O3	*O1:O4	O2:O3	O2:O4	O3:O4
	T factor	5	T1:T2	T1:T3	*T1:T4	T2:T3	T2:T4	T3:T4

*Omitted pairwise comparisons

The correct version of Table 6 is

**Table 6:** Pairwise comparison set

Level		Set	Pairwise comparison set					
Second		1	S:W	S:O	*S:T	W:O	W:T	O:T
Third	S factor	2	S1:S2	S1:S3	*S1:S4	S2:S3	S2:S4	S3:S4
	W factor	3	W1:W2	W1:W3	*W1:S4	W2:W3	W2:W4	W3:W4
	O factor	4	O1:O2	O1:O3	*O1:O4	O2:O3	O2:O4	O3:O4
	T factor	5	T1:T2	T1:T3	*T1:T4	T2:T3	T2:T4	T3:T4

*Omitted pairwise comparisons

In Table 12, the ranks of “O4 Increased demand for clarification of false information” and “T4 Demand for personal Health Record” were incorrectly formatted in boldface but should have been in regular font.

In Table 13, the column 4 header was incorrectly given as ‘Expert’s opinion’ should have been blank, and this header “Expert’s opinion” should be shifted to column 5. In addition, the “Information Quality” data in column 4 is shifted to column 5.

The correct and incorrect versions of Table 13 are shown below:

The incorrect version of Table 13

**Table 13:** Expert’s opinion in the area of evaluating online consumer information

Evaluation factors	Assessment areas	Sub-areas	Expert’s opinion	
Information Quality	Information Content	Accuracy	Fundamental quality health information	
		Diversity	-	
		Reliability	Trustworthy information	
		Understandability	Understandable / From the perspective of the people/Easy term and image for consumer/ Health information at the consumer's level/ Processing information for consumers /Health literacy	
		Up-to-date	Seasonal health information and preventive vaccinations. / Timeliness and Promptness / Timely and Appropriate Information	
		Connectivity	-	
System Quality	Design and interface	Conveniences	Ease of accessibility	Easy Accessibility / Promotion and Marketing Strategy / Ease of Accessing Information/ Seems to be Less Known/ Usability/ Portal Promotion/ Access to Consumers/ Awareness/ Website Ranking/ Portal Awareness/ Lack of Promotion
		Communicability	Search usability	-
			Readability	Text Readability is Poor
			Changes based on user behaviors	-
			Consistency of design	Menu Structure and UI Should Be Convenient
			Intuitiveness of design	UI and UX Design/ I'm not sure if there's more meaning beyond gathering a lot of information in one place
	System Operations and Management	Stability	Automation	Channels like KakaoTalk/ Automation
			Security	-
			Optimization	-
Service Quality	Service	Reactivity	The speed of questions and answers	-
			Diversity of feedback channels	Consumer Channels/ Diverse Channels/ Incorporating Consumer Opinions
			Personalization by audience	For elderly population
		Flexibility	Easy navigation	No description on the website/ Difficult to understand what the website is about
			Degree of openness	-
			accessibility	-
		Integration	Interoperability	-
			Unify information provision	Mobile App Development/ Mobile App

Lee, Bohan, & Lee, KumNoh (2022). Research on Measures for Improving Online Provision of Consumer Information. Korean Consumer Agency, from: https://www.kca.go.kr/home//sub.do?menukey=6101&mode=view&no=1003469456

The correct version of Table 13

**Table 13:** Expert’s opinion in the area of evaluating online consumer information

Evaluation factors	Assessment areas	Sub-areas		Expert’s opinion
Information quality	Information content	Accuracy		Fundamental quality health information
		Diversity		–
		Reliability		Trustworthy information
		Understandability		Understandable/From the perspective of the people/Easy term and image for consumer/Health information at the consumer’s level/Processing information for consumers/Health literacy
		Up-to-date		Seasonal health information and preventive vaccinations./Timeliness and promptness/Timely and appropriate information
		Connectivity		–
System quality	Design and interface	Conveniences	Ease of accessibility	Easy accessibility/Promotion and marketing strategy/Ease of accessing information/Seems to be less known/Usability/Portal promotion/Access to consumers/Awareness/Website ranking/Portal awareness/Lack of promotion
			Search usability	–
		Communicability	Readability	Text readability is poor
			Changes based on user behaviors	–
			Consistency of design	Menu structure and UI should be convenient
			Intuitiveness of design	UI and UX Design/I’m not sure if there’s more meaning beyond gathering a lot of information in one place
	System operations and management	Stability	Automation	Channels like KakaoTalk/Automation
			Security	–
			Optimization	–
Service quality	Service	Reactivity	The speed of questions and answers	–
			Diversity of feedback channels	Consumer channels/Diverse channels/Incorporating consumer opinions
			Personalization by audience	For elderly population
		Flexibility	Easy navigation	No description on the website/Difficult to understand what the website is about
			Degree of openness	–
			Accessibility	–
		Integration	Interoperability	–
			Unify information provision	Mobile app development/Mobile app

Lee, Bohan, & Lee, KumNoh (2022). Research on Measures for Improving Online Provision of Consumer Information. Korean Consumer Agency, from: https://www.kca.go.kr/home//sub.do?menukey=6101&mode=view&no=1003469456

The original article has been corrected.

